# Feasibility Study on Menstrual Cycles With Fitbit Device (FEMFIT): Prospective Observational Cohort Study

**DOI:** 10.2196/50135

**Published:** 2024-03-12

**Authors:** Anna-Lena Lang, Rosa-Lotta Bruhn, Maya Fehling, Anouk Heidenreich, Jonathan Reisdorf, Ifrah Khanyaree, Maike Henningsen, Cornelius Remschmidt

**Affiliations:** 1 Data4Life gGmbH Potsdam Germany; 2 Faculty of Health University Witten Herdecke Witten Herdecke Germany

**Keywords:** women’s health, menstrual cycle, premenstrual syndrome, PMS, mobile app, wearable device, sensor data, digital health

## Abstract

**Background:**

Despite its importance to women’s reproductive health and its impact on women’s daily lives, the menstrual cycle, its regulation, and its impact on health remain poorly understood. As conventional clinical trials rely on infrequent in-person assessments, digital studies with wearable devices enable the collection of longitudinal subjective and objective measures.

**Objective:**

The study aims to explore the technical feasibility of collecting combined wearable and digital questionnaire data and its potential for gaining biological insights into the menstrual cycle.

**Methods:**

This prospective observational cohort study was conducted online over 12 weeks. A total of 42 cisgender women were recruited by their local gynecologist in Berlin, Germany, and given a Fitbit Inspire 2 device and access to a study app with digital questionnaires. Statistical analysis included descriptive statistics on user behavior and retention, as well as a comparative analysis of symptoms from the digital questionnaires with metrics from the sensor devices at different phases of the menstrual cycle.

**Results:**

The average time spent in the study was 63.3 (SD 33.0) days with 9 of the 42 individuals dropping out within 2 weeks of the start of the study. We collected partial data from 114 ovulatory cycles, encompassing 33 participants, and obtained complete data from a total of 50 cycles. Participants reported a total of 2468 symptoms in the daily questionnaires administered during the luteal phase and menses. Despite difficulties with data completeness, the combined questionnaire and sensor data collection was technically feasible and provided interesting biological insights. We observed an increased heart rate in the mid and end luteal phase compared with menses and participants with severe premenstrual syndrome walked substantially fewer steps (average daily steps 10,283, SD 6277) during the luteal phase and menses compared with participants with no or low premenstrual syndrome (mean 11,694, SD 6458).

**Conclusions:**

We demonstrate the feasibility of using an app-based approach to collect combined wearable device and questionnaire data on menstrual cycles. Dropouts in the early weeks of the study indicated that engagement efforts would need to be improved for larger studies. Despite the challenges of collecting wearable data on consecutive days, the data collected provided valuable biological insights, suggesting that the use of questionnaires in conjunction with wearable data may provide a more complete understanding of the menstrual cycle and its impact on daily life. The biological findings should motivate further research into understanding the relationship between the menstrual cycle and objective physiological measurements from sensor devices.

## Introduction

An estimated 56% to 93% of women across the world experience recurrent painful periods [1-5]. Menstrual pain is debilitating for many women and has a major impact on their health-related quality of life [6-11]. One condition that can cause painful menstruation is endometriosis, which affects 10% to 15% of women of reproductive age [12]. In addition to severe pain, intermenstrual bleeding, painful periods (dysmenorrhea), painful intercourse (dyspareunia), painful bowel movements (dyschezia), and painful urination (dysuria), endometriosis can cause infertility, increase psychological distress, and can affect sexuality and relationships [12,13]. Despite its debilitating nature, it still takes around 6 to 8 years from symptom onset to diagnosis [14,15]. The gold standard for diagnosis is still laparoscopy, which is not only risky but also expensive. With the rise of gender equality movements, clinical research into menstrual health has slowly gained more attention in recent years, with researchers emphasizing the importance of timely diagnosis and treatment of menstrual cycle–related concerns and disorders [16]. Each individual has a different baseline of subjective pain experience and the availability of sufficient baseline data can enable a shift toward precision medicine in menstrual health [17]. Frequent and continuous data collection can provide an understanding of symptom variability, which is likely to be an important contributor to variability in treatment response [17]. However, traditional clinical trials to date have relied on infrequent in-person assessments and subjective retrospective data, failing to capture the daily changes in physical and mental well-being that occur over the course of the menstrual cycle [18,19]. In this regard, commercially available menstrual tracker apps offer new opportunities for research. Women can continuously track their menstrual health in digital diaries, with self-learning algorithms continuously improving predictions and educational content empowering users to increase their knowledge about menstrual health [20-22]. As a complement to subjective digital questionnaires, commercially available wearable technology can provide an easy way to collect continuous and objective real-world health data for women’s health research purposes. In the example of endometriosis, the combined collection of subjective data from digital diaries and objective wearable devices could help distinguish between “normal” menstrual pain and pain associated with endometriosis, potentially speeding up the diagnosis process and avoiding unnecessary invasive tests.

Previous research, albeit with small sample sizes, has already shown that the collection of sensor data such as step count, heart rate, and sleep duration combined with self-reported menstrual cycle data can uncover interesting correlations and advance knowledge about menstrual health [23-26]. For example, wearables are already increasingly being used to evaluate alternative contraceptive methods and to predict the fertility window [26-29]. Interesting findings from previous research using wearables include an observed lower distal skin temperature (as measured with an Oura ring) during ovulation, as well as higher heart rate in the ovulatory, mid, and late luteal phases [24,25]. While previous studies show a potential effect of physical activity on the menstrual cycle and vice versa [30-32], none of them used wearable devices for daily activity tracking during the menstrual cycle. Similarly, there is a lack of studies using wearable data to analyze sleep behavior during the menstrual cycle. Current scientific knowledge on changes in sleep behavior across the menstrual cycle is conflicting, with some studies finding a decrease in subjective and objective sleep quality during the premenstrual phase and menses [33-36], while other studies did not find such correlations [24,37]. The ongoing Apple Women’s Health Study [27], a mobile app–based longitudinal cohort study that includes both survey and sensor-based data, has not yet published results related to sensor data.

Combining subjective data from women’s health questionnaires with objective sensor data from wearables can not only facilitate cycle tracking for the everyday consumer but also allow researchers and participants alike, to gain a deeper insight into the clinical changes during the menstrual cycle. Commercially available wrist-worn sensor devices, such as the Fitbit Inspire 2 device, are thereby not only much more affordable than research-grade sensor devices, but can be nearly as accurate [38,39]. However, real-world data collection studies conducted exclusively in the home can present difficulties in terms of retention and adherence to the study protocol [19,40]. To explore the feasibility of consistently collecting wearable and questionnaire data across multiple menstrual cycles, we conducted a 12-week feasibility study with 42 participants. Using the Fitbit Inspire 2 device and digital questionnaires within the Data4Life study app, our primary objective was to refine methods for collecting authentic menstrual cycle data in a real-world setting. This included assessing participant retention rates and gathering usability feedback on their engagement with wearable devices and digital questionnaires. In addition, our study explored potential clinical correlations between wearable and questionnaire data, with the aim of uncovering potential correlations with key parameters of the menstrual cycle.

## Methods

### Study Design

This digital prospective cohort study was conducted mainly online with enrollment between December 2021 and April 2022. This study was called the Feasibility Study of Menstrual Cycles With Fitbit Device (FEMFIT). Participants were recruited by their local gynecologist at a practice in Berlin, Germany. The owner of the practice, who is also a coauthor of this study (MH), received financial incentives for recruiting participants. During recruitment, participants received a token to access the FEMFIT study in the study app. During account creation, participants provided digital informed consent to share their data for research. All participants received a free Fitbit Inspire 2 (Fitbit International Limited) as an incentive after completing the study. Participants were asked to wear the device at all times throughout the study period of 3 menstrual cycles or 12 weeks. Dropout was defined as individuals who did not provide data for more than 14 days. This cut-off was deliberately set to manage participant dropout while ensuring the inclusion of data from individuals with shorter menstrual cycles.

The primary outcome variables of this study centered on assessing the feasibility of continuous menstrual cycle data collection using the Fitbit Inspire 2 device and digital questionnaires. Specifically, we focused on participant retention rates and usability feedback regarding engagement with these tools. In addition, secondary outcome variables included exploring potential clinical correlations between the collected wearable and questionnaire data and key menstrual cycle parameters.

### Data Collection: Digital Questionnaires and Wearable Data

Prior to enrollment, participants completed a paper questionnaire to assess their digital literacy [41] (Multimedia Appendix 1).

Digital questionnaires (Multimedia Appendix 1; Figure S2 in Multimedia Appendix 2) were accessible within the app at varying intervals, with email notifications reminding participants of new questions. Email notifications reminded participants of newly available questions. At enrollment, participants provided demographic information and were asked for clinical information with a focus on women’s health, including preexisting conditions, use of hormonal contraception, cycle regularity, and the first day of the last menses (Multimedia Appendix 1). After enrollment, weekly questionnaires focused on monitoring the duration of wearable device use duration and assessing mental and physical well-being (Multimedia Appendix 1). Notably, the specific results of the mental and physical well-being assessments are not presented in this publication. Triggered by the first day of their last menstrual cycle, from day 13 to day 5 of their menstrual cycle, participants were asked about the 12 typical symptoms of premenstrual syndrome (PMS): seclusion, irritability, swelling, anger, weight gain, joint pain, headache, confusion, dejection, bloating, anxiety, and tenderness [42]; participants were also asked about the severity of their bleeding. If a new menstrual cycle had begun, the first day of bleeding triggered the new cadence for all future questionnaires. Data from these daily questionnaires were used for correlation with the wearable data collected simultaneously.

In addition to the digital questionnaires, the Fitbit device recorded 3 parameters daily: average resting heart rate, total steps, and total sleep time. For this study, average resting heart rate was rounded to the nearest 5 beats per minute, sleep time was rounded to the nearest 10 minutes, and step count was rounded to the nearest 100 steps. This generalization was made to protect the anonymity of participants when analyzing deidentified donation records in this small feasibility study.

### Definitions

The relevant phases of the menstrual cycle were defined as follows: the menses phase (MP; days 1 to 5), the ovulation day (OD; day 1 of the next MP minus 14) [43], the follicular phase (day 1 of MP to OD minus 1), the luteal phase (OD plus 1 to the menses), and the midluteal phase (OD plus 3 to OD plus 9).

In our analysis of PMS, only PMS symptoms from the days after OD to the end of MP were considered. Severe PMS was defined as a symptom severity rating of 3, equivalent to severe, on the visual analog scale. Participants were included in the severe PMS group if they reported severe PMS symptoms in at least 2 cycles. Participants were included in the no or low PMS group if they never reported PMS symptom severity higher than 1 on a severity scale from 0 to 3.

### Data Storage and Wearable Data Integration

The nonprofit organization Data4Life provided the research infrastructure (study application, data storage, and analysis platform) for this study. All research data was stored in Data4Life’s secure research environment on servers in Germany. Data4Life is certified by the German Federal Office for Information Security (BSI). For participants who allowed the use of cookies, we were able to analyze user behavior using the General Data Protection Regulation–compliant business analytics tool Matomo (Matomo; data stored in Europe only). In the web app, after entering their study token and providing digital study consent, participants were asked to connect to Fitbit to allow access to their wearable data via the Fitbit web application programming interface (API) [44]. After completing the OAuth 2.0 Authorization Code Grant Flow [45], the API refresh token was stored in end-to-end encrypted form for subsequent use for the duration of their participation in the study or until they withdrew their consent. This allowed the study’s wearable parameters to be retrieved client-side via the respective activity and sleep endpoints, and stored in end-to-end encrypted form as Fast Healthcare Interoperability Resources Standard for Trial Use, version 3, [46] observation records each time participants logged into the web app to complete their questionnaires. As the API returns time series data over a few days, data gaps between donations were filled by comparing previously stored records with the data points returned by the API.

### Statistical Analysis

All analyses were performed on the Data4Life analytics platform on a jupyterhub notebook running Python (version 3.10.4), using *pandas* (version 1.4.2), *matplotlib* (version 3.5.2), *seaborn* (version 0.11.2), and *numpy* (version 1.22.4). Descriptive statistics were used to report details of the study cohort, retention, and adherence measures. Continuous values were reported as mean with SD or median with IQR; categorical values were reported as numbers with percentages. For comparative analyses of clinical outcomes, we used the Mann-Whitney test [47] for nonnormally distributed data and the 2-tailed *t* test [48] for normally distributed data.

### Ethical Considerations

The study was approved by the ethics committee of the Berlin Chamber of Physicians (Eth-11/22). Registration was open for people aged 18 years and older. All participants provided digital informed consent for study participation. Participation was voluntary. Participants were allowed to keep the Fitbit Inspire 2 device after the study ended. Email and password were required to log in to the study app as well as two-factor authentication via phone. Participants could access all study content through a web app on both desktop and mobile devices. All study data was end-to-end encrypted and pseudonymized. Only authorized researchers were provided access to the data on the Data4Life analytics platform. Data was stored exclusively on Data4Life data centers in Germany. Based on IT-Grundschutz (ISO 27001), Data4Life is certified by the German Federal Office for Information Security (BSI).

## Results

### Study Setting and Participants

**Figure 1:**
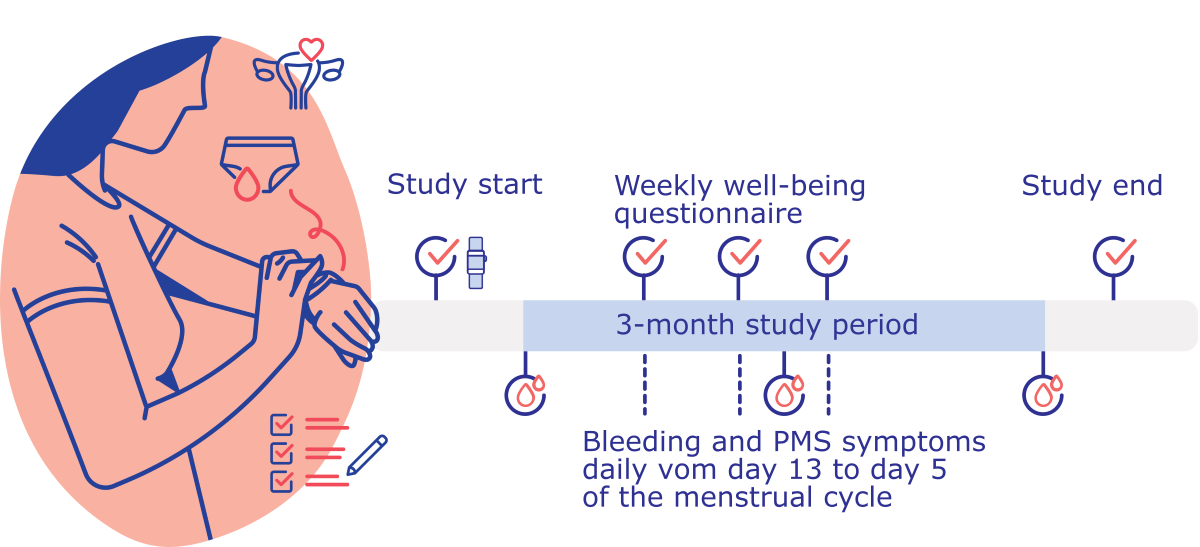
Feasibility Study of Menstrual Cycles With Fitbit Device (FEMFIT) study concept. The FEMFIT study on collecting combined wearable and questionnaire data on the menstrual cycle was carried out online. Self-reporting of questionnaire data in a study app was accompanied by the collection of wrist-worn sensor data from Fitbit Inspire 2 devices. Weekly questionnaires asked about physical and mental well-being. From day 13 to day 5 of the menstrual cycle, participants answered daily questionnaires on bleeding severity and PMS symptoms. PMS: premenstrual syndrome.

This feasibility study of combined wearable and questionnaire data collection on the menstrual cycle was conducted online with self-reporting of questionnaire data in a study app accompanied by the collection of wrist-worn sensor data from Fitbit Inspire 2 devices. A total of 42 cisgender women were recruited from December 2021 to April 2022 at a gynecological practice in Berlin and agreed to provide menstrual cycle data for 12 weeks (Figure 1).

One participant never requested a wearable device and therefore dropped out of the study before creating an account in the study app. Three participants never donated any questionnaire or wearable data (Figure S1 in Multimedia Appendix 2), leaving data from 38 participants who started to donate data in the study app. Of these, a total of 5 participants withdrew from the study within the first 2 weeks of study entry, 3 of whom never donated any wearable data, leaving valuable data from 33 participants for further analysis. Active participants (n=33) were on average 24.2 (SD 3.0) years old and predominantly identified as female, with 1 individual identifying as diverse (Table 1). Five participants (15.1%) reported preexisting mental health problems (Table 1). In terms of women’s health issues, 2 (6.1%) participants reported a history of chlamydia infection and 1 person (3%) reported a history of human papillomavirus infection. At enrollment, the median time since the last menstruation was 17 (IQR 12-26) days.

**Table 1:** Details on study participants (n=33).

Demographics of active study participants		Values
Age (years), mean (SD)		24.2 (3.0)
**Gender, n (%)**		
	Female	32 (97)
	Diverse	1 (3)
**Preexisting health condition, n (%)**		
	Chlamydia	2 (6.1)
	HPV^a^	1 (3)
	Mental health	5 (15.1)
	Hypothyroidism	1 (3)
	Hyperthyroidism	1 (3)
**Cycle regularity, n (%)**		
	Irregular	2 (6.1)
**Hormonal contraception, n (%)**		
	Yes	4 (12.1)
Days since last period, median (IQR)		17 (12-26)

^a^HPV: human papillomavirus.

### High Degree of Digital Literacy of Study Participants

Of the 42 people initially recruited, 25 (59.5%) people reported that they had never worn a wearable device before. Of those who had experience wearing a sensor device, 5 had worn an Apple Watch, and other devices worn included Fitbit (n=2), Garmin (n=1), Samsung (n=1), and Denver (n=1). The majority of participants (26/42, 61.9%) regularly used apps on their smartphones to track their food intake, physical activity, or menstrual cycle. The most commonly used menstrual cycle tracking apps were Flo (9/26, 34.6%) and Clue (4/26, 15.3%). At the start of the study, a standardized digital literacy questionnaire [41] showed that the participants were highly digitally literate with an average score of 37.1 (SD 3.1) out of 48.

### Increased User Engagement After Early Dropouts

Across all 41 participants who enrolled in the study and logged into the study app, the mean time spent in the study (measured from enrollment to last weekly or daily questionnaire delivery) was 63.3 (SD 33.0) days The reported reasons for not completing the study were too great a time commitment (n=1), a high technical barrier (n=2), and technical difficulties that made it impossible to complete the study (n=3).

**Figure 2:**
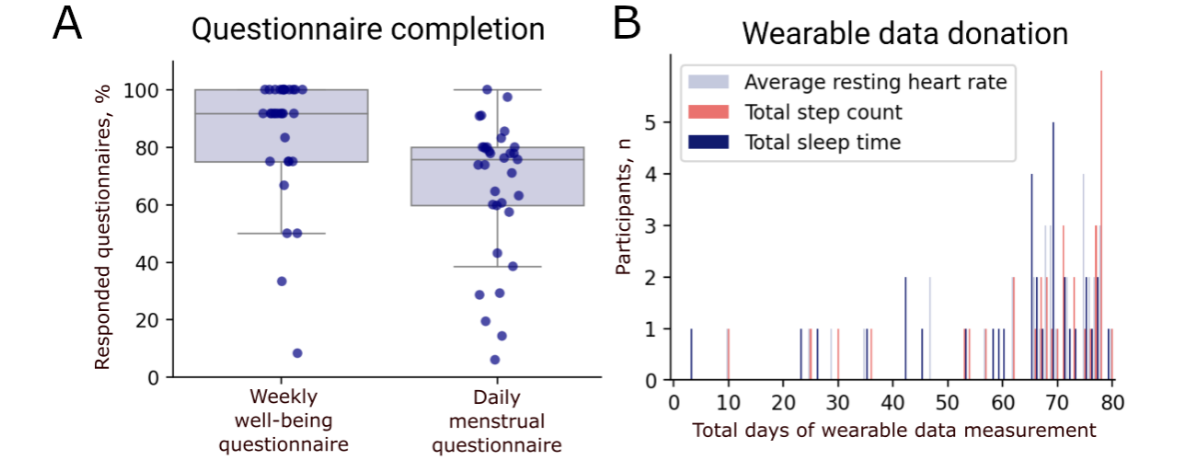
Participant engagement. (A) Questionnaire completion rates of weekly and daily questionnaires. Questionnaires on physical and mental well-being were filled out once per week, whereas questions on premenstrual symptoms and bleeding severity were filled out daily from day 13 to day 5 of the menstrual cycle. The box plots show the median and IQR, with whiskers reflecting minimum and maximum values for outliers. Dots represent all data points. (B) Bar plot visualizing the total days of wearable data donation during the study period per participant. Bars are color-coded by the parameter assessed using the Fitbit Inspire 2 wearable device.

Among participants who remained in the study for more than 14 days (n=33), questionnaire completion rates were high. The weekly physical and mental well-being questionnaires were completed on average 10.2 times (SD 2.6) out of 12, while 69.7% (n=23) of active participants completed at least 11 of the 12 weekly questionnaires. An average of 65.8% (SD 24.1%) of all questionnaires on menstrual bleeding and PMS symptoms, which were asked daily from day 13 to day 5 of the menstrual cycle, were completed (Figure 2A). Overall, 66.7% (n=22) of participants reported starting dates of at least 3 menstrual cycles, with a median cycle length of 28.0 days (IQR 24-31), and we collected data from a total of 50 complete ovulatory cycles. In general, logins to our study app occurred mostly after email notifications (Figure S3 in Multimedia Appendix 2), suggesting that frequent study reminders may increase retention.

The average number of days wearable data were provided was 65.2 (SD 17.0) days for step count, 64.2 (SD 17.1) days for heart rate, and 60.5 (SD 17.8) days for sleep time (Figure 2B). There were difficulties in collecting continuous wearable data over the entire study period, particularly for sleep time. None of the 33 active participants provided consecutive wearable data over the entire study period. The average number of consecutive days of data donation was 36.4 (SD 25.6) days for sleep time, 38.6 (SD 31.4) days for heart rate, and 52.6 (SD 28.3) days for step count. Wearable data was provided consecutively for at least 50 days by 11 participants and for more than 70 days by 3 participants. In the weekly study questionnaire, participants reported that they were wearing the device continuously for an average of 9 (SD 2.9) weeks out of 12. When participants reported that they did not wear the device continuously, off-times were stated to be longer than 3 hours per day.

### Fitbit Device and Study App Supported Subjective Health Monitoring

At the end of the study, 22 participants completed questions about their study experience. Most participants reported that the Fitbit device was very easy to use (n=22; mean rating 1.6, SD 0.6; 1=very easy, 5=very challenging) and did not interfere with their daily life (n=22; mean rating 1.2, SD 2; 1=very comfortable, 5=very disturbing). One person found wearing the Fitbit device at night very uncomfortable, whereas overall it was not perceived to be bothersome at night (n=22; mean rating 2.2, SD 1).

The majority of participants completing the end-of-study questionnaire (13/22, 59%) stated that wearing the device increased their physical activity over the study period. Step count data from all participants over the study period thereby showed an average step count of 9987 (SD 3856) steps per day in the last 2 weeks of study participation compared with an average of 9301 (SD 4076) steps per day in the first 2 weeks of study participation, but this observation was not significant. Overall, participants did not feel that the sensor tracking changed their sleep behavior (13/22, 59%). Five participants reported that wearing the device and answering the questionnaires made them feel stressed, while 9 participants reported that wearing the Fitbit device made them feel more in control. For 15 participants (67.9%), the study helped them to better associate symptoms such as mood swings, headaches, and pelvic pain with a particular phase of their menstrual cycle. Overall, the study seemed to improve participants’ health awareness, and 21 of the 22 participants who completed the final questionnaire said they would take part in a similar study again.

### Digital Symptom Questionnaires Revealed Patterns of PMS Symptoms Across the Menstrual Cycle

PMS-related symptoms and their severity on a scale of 0 to 3 were only assessed on potential PMS days during the luteal phase and menses. The majority of participants (22/33, 66.7%) reported severe PMS symptoms on at least 1 day of the menstrual cycle for at least 2 cycles. Severe PMS was reported by 18.2% (n=6) of participants in at most one of the cycles recorded in our study, and only 15.2% (n=5) did not experience any severe PMS symptoms during the study period.

**Figure 3:**
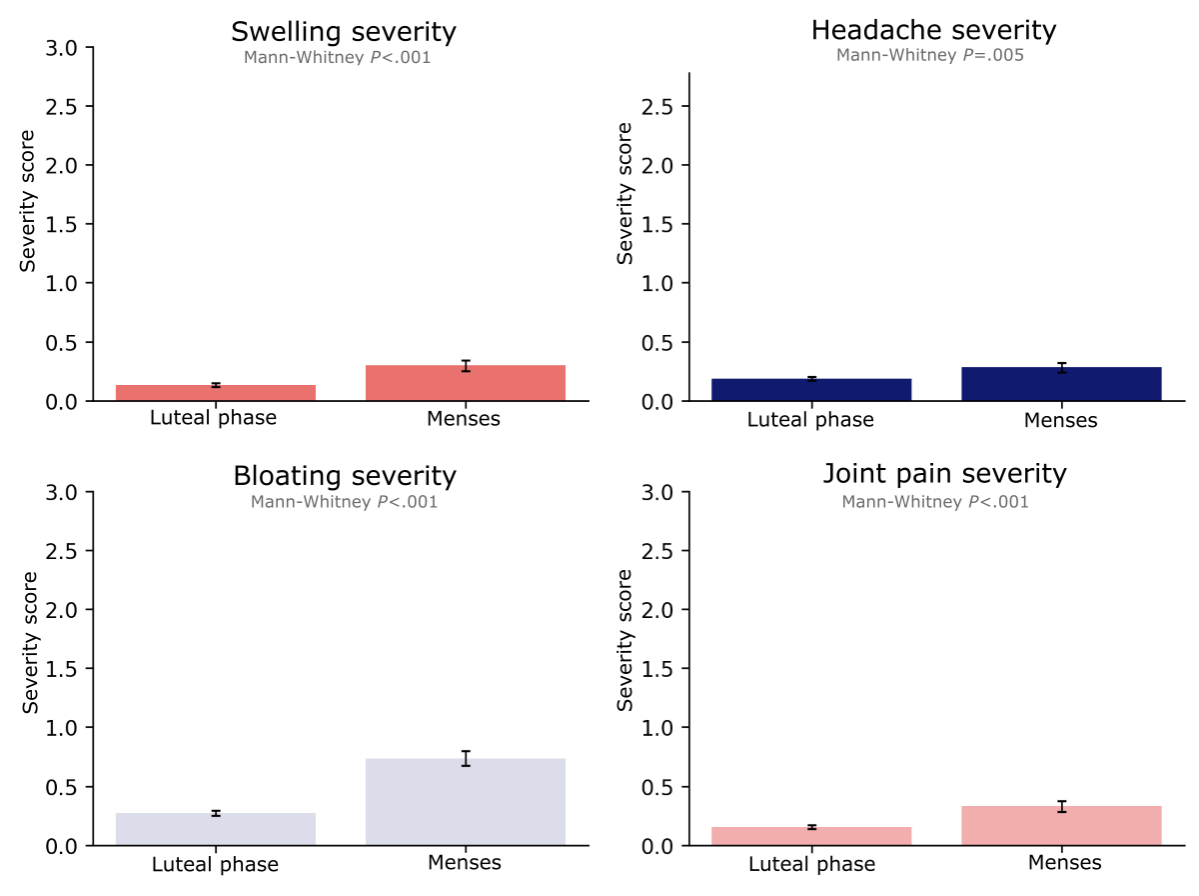
Symptom severity over the menstrual cycle. Bar plots show the mean rating for symptom severity on a scale from 0 to 3 (0=no symptoms to 3=very strong symptom severity). Error bars are the SE. Mann-Whitney *P* values comparing groups are shown for each plot.

In terms of mental health, dejection (average score 0.5, SD 0.9) and seclusion (average score 0.3, SD 0.7) were among the most commonly reported symptoms during the menses. The data suggested a notable impact on participants’ mental health during the menses, with higher severity of seclusion (Mann-Whitney *P*=.03), dejection (Mann-Whitney *P=*.02), and irritability (Mann-Whitney *P=*.01) reported during the menses compared with the luteal phase (Figure S4 in Multimedia Appendix 2). Reported levels of anxiety, confusion, anger, and tenderness did not differ substantially between the luteal phase and menses (Figure S4 in Multimedia Appendix 2). In contrast to mental health symptoms, reported physical symptoms during menses were more severe. Participants experienced a significantly higher severity of bloating during the menses (mean 0.7, SD 1.1) compared with the luteal phase (mean 0.3, SD 0.7; Mann-Whitney *P*<.001; Figure 3). Similarly, the swelling was reported to be more severe during the menses (mean 0.3, SD 0.8; luteal phase: mean 0.1, SD 0.5; Mann-Whitney *P*<.001). In addition, participants reported more severe headaches during the menses compared with the luteal phase (mean 0.38, SD 0.7; luteal phase: mean 0.2, SD 0.67; Mann-Whitney *P*=.005) and more bothersome joint pain during the menses (mean 0.3, SD 0.8; luteal phase: mean 0.2, SD 0.5; Mann-Whitney *P*<.001). In terms of reported weight gain, there was a trend toward increased weight gain during the menses, although this increase did not reach statistical significance (Figure S4 in Multimedia Appendix 2).

### Combined Questionnaire and Wearable Data Delivered Interesting Insights Into the Menstrual Cycle

Participants provided a total of 23,424 symptom reports, with a total of 2468 symptoms reported in the daily questionnaires, which were administered during the luteal phase and menses. Heart rate sensor data was collected on a total of 2118 days, step count on 2153 days, and sleep time on 1996 days.

**Figure 4:**
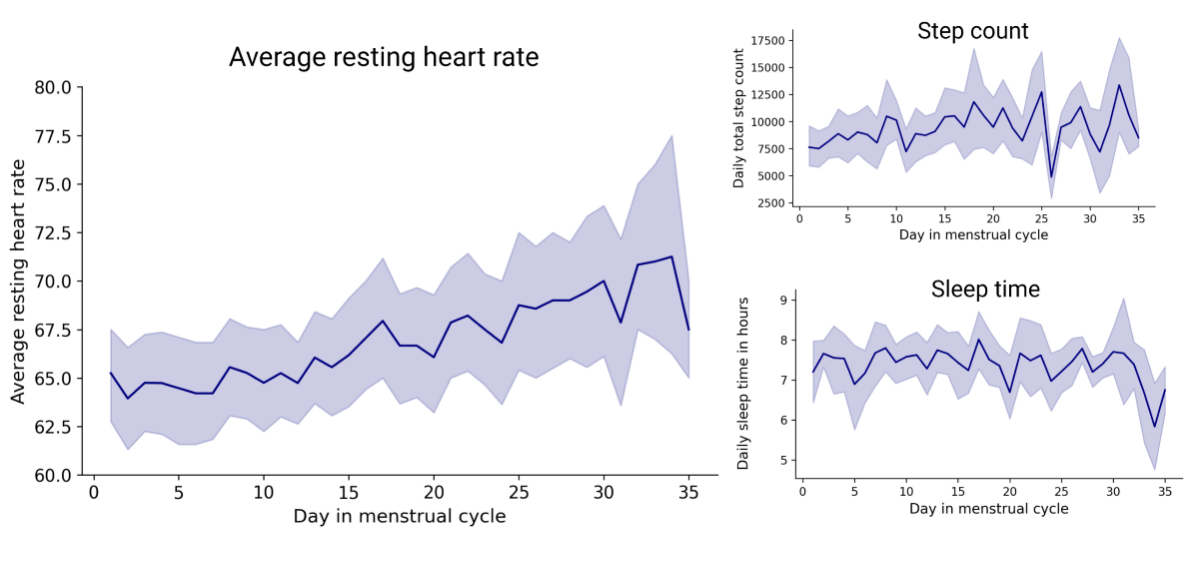
Physical parameters measured by wearable devices over the menstrual cycle. Progression of daily average resting heart rate, step count, and sleep time over the menstrual cycle across all active study participants. Only cycles with a maximum length of 35 days were included. The dark lines show the mean and the shadowed lines show the 95% CI. The average resting heart rate was highest in the mid and late luteal phase relative to the time from menses to ovulation (Mann-Whitney *P*<.001); no significant difference was seen for sleep time or step count.

The average resting heart rate over the entire study period was 66.7 (SD 6.7). Participants slept an average of 7.3 (SD 1.6) hours while wearing the Fitbit device. With an average of 10,184 (SD 6120) steps per day, the FEMFIT cohort was quite physically active, with some participants walking >20,000 steps per day (153 days from 21 participants recorded with >20,000 steps; Figure S5 in Multimedia Appendix 2). When examining correlations between sensor data and questionnaire data on the menstrual cycle, we observed that mean resting heart rate appeared to be highest in the mid and late luteal phases relative to the time between menses and ovulation (Mann-Whitney *P<*.001; Figure 4, Figure S6 in Multimedia Appendix 2). We did not observe any significant differences in the change in step count (Mann-Whitney *P=*.72) or sleep time (Mann-Whitney *P=*.58) between the follicular and luteal phases (Figure 4).

In an exploratory analysis of the wearable data across participants with different PMS severity, participants with severe PMS (n=22) walked significantly fewer steps (mean daily steps10,283, SD 6277) during the luteal phase and menses compared with participants with no or low PMS (n=5; mean 11,694, SD 6458; Mann-Whitney *P*<.001). There was no significant difference in sleep duration (Mann-Whitney *P=*.18) or heart rate with PMS severity (Mann-Whitney *P=*.72).

To investigate whether hormonal contraception had an effect on step count, sleep time, or heart rate, we compared 4 participants using hormonal contraception with 29 participants not using hormonal contraception. Participants on hormonal contraception appeared to be less active (mean step count 7400, SD 4433; no hormonal contraception: mean 9800, SD 6203; Mann-Whitney *P<*.001). The average daily resting heart rate was significantly higher in the hormonal contraceptive group (mean 68.2, SD 7.7 vs mean 66.6, SD 6.6; Mann-Whitney *P<*.001). There was no difference in sleep behavior between the 2 groups.

## Discussion

### Principal Findings

To date, only a few studies have assessed the menstrual cycle using both objective wearable and subjective questionnaire data [23,27,29,49,50]. Previous research has demonstrated potential effects of physical activity on the menstrual cycle and vice versa [30-32]. However, none of these studies used wearable devices to monitor daily activity levels, and there is a gap in studies using wearable data to examine sleep patterns across the menstrual cycle. Recognizing that participant retention is a significant challenge in digital cohort studies [19,40], our feasibility study was designed to primarily assess the effectiveness of study participation and adherence to the study protocol, highlighting challenges and opportunities for future large-scale studies in women’s health. Secondarily, we focused on potential biological outcomes.

Our cohort was a group of young, mostly digitally literate, cisgender women with regular cycle lengths. Overall, retention was good among participants who remained in the study beyond the second week. The observed dropout rate of 21.4% (n=9) within the first 2 weeks of the study is consistent with rates reported in other digital cohort studies [51,52], and the overall median retention of 81.0 days was remarkably high [52]. Nevertheless, these dropouts may have introduced selection bias. Among the 5 dropouts who provided demographic data, we did not identify any discernible demographic patterns in relation to retention. The mean age of the dropouts was 24.8 (SD 3.2) years, which was not significantly different from the rest of the cohort. None of the dropouts had preexisting medical conditions and only 1 reported menstrual irregularity. In future large-scale studies, it would be advisable to collect demographic information directly at recruitment (in our case when the wearable device was distributed) to ensure comprehensive data on dropouts. This may contribute to a better understanding of the potential retention patterns and help to address selection bias. Among those who dropped out early, technical barriers were the most commonly reported problem. Although our study team provided access to technical support, future studies could potentially reduce dropout rates by further reducing barriers to accessing technical support or by conducting active outreach. The young age of participants, such as our cohort, is also known to be associated with lower retention [19,52]. Targeted engagement strategies such as increased outreach and communication with younger participants could counteract this [19]. For large-scale studies, continuous monitoring of data donations in an ongoing study can facilitate real-time strategies such as increased outreach to engage inactive participants, reduce dropouts, and ensure data completeness [19]. In 2 similar studies using sensor devices to investigate physiology surrounding the menstrual cycle [23,25], retention was generally not an issue, but retention efforts were not further highlighted in the publications.

In addition to the potential bias introduced by study dropouts, ensuring high data completeness is critical for investigating temporal patterns in menstrual cycles. Similar to our study, Maijala et al [23] reported issues with continuous data contribution across consecutive days. In particular, issues related to participant retention and data completeness could potentially undermine the integrity of the results. In our study, this was particularly challenging for sleep data. To mitigate these concerns, several strategies can be considered for future research. The comfort of wearable devices should be a priority in study design, as some participants in our study found the devices too uncomfortable. Data completeness may be further increased if a clear purpose for data collection is visible to participants, for example, having the clinical condition of interest in the study [52,53]. As this was a feasibility study without a specific disease focus, we expect retention and data completeness to be higher when applying a similar study setting to a clinical condition with a high disease burden, such as endometriosis [54]. Recruitment strategies should also be tailored to improve data completeness. Although participants in our study were highly digitally literate, the vast majority had never worn a wearable device before. Recruiting participants who already have a sensor device and are accustomed to wearing it on a daily basis may increase data completeness. However, such targeted recruitment would introduce additional bias, as sensor devices are mostly owned by individuals from high-income households [55]. Although some participants in our study felt stressed by wearing the sensor devices, positive prior experience with wearing sensor devices as a selection criterion for study participation may further increase data completeness. In our study, participants received the wearable device as a free gift at the start of the study, and the incentive was not dependent on their questionnaire completion rate or the duration of wearing the device. Future studies may benefit from offering incentives upon successful completion of the entire study, thereby encouraging full participation [52,56]. Increasing compensation beyond just the Fitbit device could further improve retention [52]. In terms of questionnaire data completeness, it may be beneficial to reduce the number of daily questions. In our study, completion rates for daily questionnaires were lower than for weekly questionnaires, and 1 of the 9 people who dropped out within the first 2 weeks reported that the study was too time-consuming. These strategies can potentially help to improve participant retention and ensure more comprehensive and reliable data.

Despite challenges related to data completeness, our study successfully demonstrated the technical feasibility and value of combining wearable and questionnaire data. The information derived from the digital questionnaires helped to map the sensor data to the respective day of the menstrual cycle and to correlate the objective physiological data with the subjectively reported symptom data from the study app, leading to interesting biological insights. The average resting heart rate measured by wrist-worn wearable sensors changed over the course of the menstrual cycle, with an increase in the mid and late luteal phases compared with the menses. This observation supports the findings of a larger study by Shilaih et al [25], where the pulse rate measured using laboratory standard photoplethysmography technology showed a peak in the midluteal phase. Another study with a smaller sample size similarly found increased heart rate (measured during sleep) in the mid and late luteal phases compared with menses and ovulation [24]. In our feasibility study, we also found that people with more severe PMS symptoms had lower daily step counts. Due to the observational nature of our study, we cannot establish causality. This finding may suggest that individuals with more severe PMS symptoms may exercise less due to the impact of the symptoms on their physical activity. Alternatively, this finding may be consistent with the existing literature, where increased physical activity has been found to reduce PMS symptoms [57-62]. In attempting to fill the research gap regarding sleep patterns across the menstrual cycle, we did not make any notable discoveries when comparing sleep duration between different phases of the menstrual cycle, across PMS severity, or when comparing individuals using hormonal contraception with those who did not. It is worth noting that there were more issues with data completeness for sleep data compared with daytime data, highlighting the need for improved retention strategies to motivate participants to wear the devices consistently at night. Although of very small sample size, the observed differences in heart rate and physical activity in participants using hormonal contraception (n=4) also provided an interesting starting point for future investigation. These observations show that sensor devices may offer a great opportunity to study physical changes over the menstrual cycle and suggest that further investigation on a larger scale may be valuable. Overall, it is important to interpret the biological findings of this feasibility study with caution, given the limitations of the small sample size and issues with data completeness.

### Limitations

Apart from the interesting results of this feasibility study, this study has several limitations that should be taken into account when interpreting the findings.

First, the study was conducted as a feasibility study with a small sample size. Any biological interpretations made from such a small sample size must be taken with caution and merely represent an interesting starting point for future large-scale research. Our cohort was also not representative, recruited from only 1 recruitment site, and lacked ethnic diversity.

We further had limitations in data accuracy. The amount of wearable data we collected was limited to 3 aggregated parameters, which severely limited our ability for quality filtering. As we did not collect continuous wearable data throughout the day, but rather one aggregated data point for each measurement of interest, we had no objective insight into how long the device was worn each day and relied only on subjective reporting of wear time in the weekly questionnaires. Not wearing the device consistently or correctly could have resulted in inaccurate data.

Furthermore, the study relied on self-reported questionnaire data, which may introduce reporting bias and errors in data collection. In addition, the wrist-worn wearable device used in our study may not be as accurate as research-grade devices in tracking changes in physiological parameters related to the menstrual cycle. One must consider potential confounding factors that may affect the measurement accuracy of the sensor devices such as body sweat and unusual movements [63].

Regarding the medical accuracy of possible correlations between wearable data and specific phases of the menstrual cycle, the design of our study only allowed the day of ovulation to be estimated based on the total cycle length. In future studies, additional at-home ovulation tests could ensure greater accuracy in assessing the different phases of the menstrual cycle [25].

### Conclusions

In conclusion, this study demonstrated that an app-based approach to collecting combined wearable and questionnaire data on the menstrual cycle is technically feasible and provides interesting biological insights. Study participants had a high level of digital literacy, which may have supported adherence to the study protocol. With 9 of 42 participants dropping out within the first 2 weeks, engagement efforts at baseline would need to be improved in future, larger studies to ensure long-term adherence to the study protocol. To further improve the quality and applicability of such large-scale studies, challenges with data completeness need to be addressed. This could include using more frequent reminders for study participation, using more user-friendly wearable technology, or providing incentives to encourage participants to maintain consistent data contributions over the entire study. Although the sample size was small, we discovered an increase in average resting heart rate over the menstrual cycle and found differences in step count by PMS symptom severity in our cohort. Ultimately, using digital studies to research the menstrual cycle can be feasible and has the potential to improve our understanding of women’s health and inform the development of personalized health care approaches.

## Appendix

Multimedia Appendix 1 Study questionnaires.

Multimedia Appendix 2 Supplementary figures.

## Abbreviations

API: application programming interface
FEMFIT: Feasibility Study of Menstrual Cycles With Fitbit Device
MP: menses phase
OD: ovulation day
PMS: premenstrual syndrome

## Data Availability

The data sets generated or analyzed during this study are available from the corresponding author on reasonable request.

## References

[1] Santos LBD, Barbosa IR, de Macedo Dantas TH, Araujo CM, Dantas JH, Ferreira CWS, da Câmara SMA, Dantas D (2022). Prevalence of primary dysmenorrhea and associated factors in adult women. Rev Assoc Med Bras (1992).

[2] Hadjou OK, Jouannin A, Lavoue V, Leveque J, Esvan M, Bidet M (2022). Prevalence of dysmenorrhea in adolescents in France: results of a large cross-sectional study. J Gynecol Obstet Hum Reprod.

[3] Bakhsh H, Algenaimi E, Aldhuwayhi R, AboWadaan M (2022). Prevalence of dysmenorrhea among reproductive age group in Saudi women. BMC Womens Health.

[4] Karout S, Soubra L, Rahme D, Karout L, Khojah HMJ, Itani R (2021). Prevalence, risk factors, and management practices of primary dysmenorrhea among young females. BMC Womens Health.

[5] Durand H, Monahan K, McGuire BE (2021). Prevalence and impact of dysmenorrhea among university students in Ireland. Pain Med.

[6] Ju H, Jones M, Mishra G (2014). The prevalence and risk factors of dysmenorrhea. Epidemiol Rev.

[7] Schoep ME, Nieboer TE, van der Zanden M, Braat DDM, Nap AW (2019). The impact of menstrual symptoms on everyday life: a survey among 42,879 women. Am J Obstet Gynecol.

[8] Pogodina A, Dolgikh O, Astakhova T, Klimkina J, Khramova E, Rychkova L (2022). Health-related quality of life and menstrual problems in adolescents. J Paediatr Child Health.

[9] Sharma A, Taneja DK, Sharma P, Saha R (2008). Problems related to menstruation and their effect on daily routine of students of a medical college in Delhi, India. Asia Pac J Public Health.

[10] Ballagh SA, Heyl A (2008). Communicating with women about menstrual cycle symptoms. J Reprod Med.

[11] Barnard K, Frayne SM, Skinner KM, Sullivan LM (2003). Health status among women with menstrual symptoms. J Womens Health (Larchmt).

[12] Parasar P, Ozcan P, Terry KL (2017). Endometriosis: epidemiology, diagnosis and clinical management. Curr Obstet Gynecol Rep.

[13] Sinaii N, Plumb K, Cotton L, Lambert A, Kennedy S, Zondervan K, Stratton P (2008). Differences in characteristics among 1,000 women with endometriosis based on extent of disease. Fertil Steril.

[14] Ghai V, Jan H, Shakir F, Haines P, Kent A (2020). Diagnostic delay for superficial and deep endometriosis in the United Kingdom. J Obstet Gynaecol.

[15] Nnoaham KE, Hummelshoj L, Webster P, d'Hooghe T, de Cicco Nardone F, de Cicco Nardone C, Jenkinson C, Kennedy SH, Zondervan KT (2011). Impact of endometriosis on quality of life and work productivity: a multicenter study across ten countries. Fertil Steril.

[16] Hennegan J, Winkler IT, Bobel C, Keiser D, Hampton J, Larsson G, Chandra-Mouli V, Plesons M, Mahon T (2021). Menstrual health: a definition for policy, practice, and research. Sex Reprod Health Matters.

[17] Izmailova ES, Wagner JA, Perakslis ED (2018). Wearable devices in clinical trials: hype and hypothesis. Clin Pharmacol Ther.

[18] Snyder M, Zhou W (2019). Big data and health. Lancet Digit Health.

[19] Zhang Y, Pratap A, Folarin AA, Sun S, Cummins N, Matcham F, Vairavan S, Dineley J, Ranjan Y, Rashid Z, Conde P, Stewart C, White KM, Oetzmann C, Ivan A, Lamers F, Siddi S, Rambla CH, Simblett S, Nica R, Mohr DC, Myin-Germeys I, Wykes T, Haro JM, Penninx BWJH, Annas P, Narayan VA, Hotopf M, Dobson RJB (2023). Long-term participant retention and engagement patterns in an app and wearable-based multinational remote digital depression study. NPJ Digit Med.

[20] Li K, Urteaga I, Wiggins CH, Druet A, Shea A, Vitzthum VJ, Elhadad N (2020). Characterizing physiological and symptomatic variation in menstrual cycles using self-tracked mobile-health data. NPJ Digit Med.

[21] Wheeler MV, Tabor VH, Teel K, LaVigne M (2015). The science of your cycle: evidence-based app design. Clue.

[22] Hantsoo L, Rangaswamy S, Voegtline K, Salimgaraev R, Zhaunova L, Payne JL (2022). Premenstrual symptoms across the lifespan in an international sample: data from a mobile application. Arch Womens Ment Health.

[23] Maijala A, Kinnunen H, Koskimäki H, Jämsä T, Kangas M (2019). Nocturnal finger skin temperature in menstrual cycle tracking: ambulatory pilot study using a wearable Oura ring. BMC Womens Health.

[24] Alzueta E, de Zambotti M, Javitz H, Dulai T, Albinni B, Simon KC, Sattari N, Zhang J, Shuster A, Mednick SC, Baker FC (2022). Tracking sleep, temperature, heart rate, and daily symptoms across the menstrual cycle with the Oura ring in healthy women. Int J Womens Health.

[25] Shilaih M, Clerck VD, Falco L, Kübler F, Leeners B (2017). Pulse rate measurement during sleep using wearable sensors, and its correlation with the menstrual cycle phases, a prospective observational study. Sci Rep.

[26] Goodale BM, Shilaih M, Falco L, Dammeier F, Hamvas G, Leeners B (2019). Wearable sensors reveal menses-driven changes in physiology and enable prediction of the fertile window: observational study. J Med Internet Res.

[27] Mahalingaiah S, Fruh V, Rodriguez E, Konanki SC, Onnela JP, de Figueiredo Veiga A, Lyons G, Ahmed R, Li H, Gallagher N, Jukic AMZ, Ferguson KK, Baird DD, Wilcox AJ, Curry CL, Suharwardy S, Fischer-Colbrie T, Agrawal G, Coull BA, Hauser R, Williams MA (2022). Design and methods of the Apple Women's Health Study: a digital longitudinal cohort study. Am J Obstet Gynecol.

[28] Piwek L, Ellis DA, Andrews S, Joinson A (2016). The rise of consumer health wearables: promises and barriers. PLoS Med.

[29] Zhu TY, Rothenbühler M, Hamvas G, Hofmann A, Welter J, Kahr M, Kimmich N, Shilaih M, Leeners B (2021). The accuracy of wrist skin temperature in detecting ovulation compared to basal body temperature: prospective comparative diagnostic accuracy study. J Med Internet Res.

[30] Pearce E, Jolly K, Jones LL, Matthewman G, Zanganeh M, Daley A (2020). Exercise for premenstrual syndrome: a systematic review and meta-analysis of randomised controlled trials. BJGP Open.

[31] Dehnavi ZM, Jafarnejad F, Goghary SS (2018). The effect of 8 weeks aerobic exercise on severity of physical symptoms of premenstrual syndrome: a clinical trial study. BMC Womens Health.

[32] Vaghela N, Mishra D, Sheth M, Dani VB (2019). To compare the effects of aerobic exercise and yoga on premenstrual syndrome. J Educ Health Promot.

[33] Romans SE, Kreindler D, Einstein G, Laredo S, Petrovic MJ, Stanley J (2015). Sleep quality and the menstrual cycle. Sleep Med.

[34] Baker FC, Driver HS (2004). Self-reported sleep across the menstrual cycle in young, healthy women. J Psychosom Res.

[35] Van Reen E, Kiesner J (2016). Individual differences in self-reported difficulty sleeping across the menstrual cycle. Arch Womens Ment Health.

[36] Hachul H, Andersen ML, Bittencourt LRA, Santos-Silva R, Conway SG, Tufik S (2010). Does the reproductive cycle influence sleep patterns in women with sleep complaints?. Climacteric.

[37] Li DX, Romans S, De Souza MJ, Murray B, Einstein G (2015). Actigraphic and self-reported sleep quality in women: associations with ovarian hormones and mood. Sleep Med.

[38] Chevance G, Golaszewski NM, Tipton E, Hekler EB, Buman M, Welk GJ, Patrick K, Godino JG (2022). Accuracy and precision of energy expenditure, heart rate, and steps measured by combined-sensing fitbits against reference measures: systematic review and meta-analysis. JMIR Mhealth Uhealth.

[39] Nissen M, Slim S, Jäger K, Flaucher M, Huebner H, Danzberger N, Fasching PA, Beckmann MW, Gradl S, Eskofier BM (2022). Heart rate measurement accuracy of Fitbit Charge 4 and Samsung Galaxy Watch Active2: device evaluation study. JMIR Form Res.

[40] Druce KL, Dixon WG, McBeth J (2019). Maximizing engagement in mobile health studies: lessons learned and future directions. Rheum Dis Clin North Am.

[41] Karrer K, Glaser C, Clemens C, Bruder C (2009). Technikaffinität erfassen—der Fragebogen TA-EG. Der Mensch im Mittelpunkt technischer Systeme. 8. Berliner Werkstatt Mensch-Maschine-Systeme.

[42] Keye WR (2004). Premenstrual Syndrome (PMS). Encycl Endocr Dis.

[43] Lenton EA, Landgren BM, Sexton L (1984). Normal variation in the length of the luteal phase of the menstrual cycle: identification of the short luteal phase. Br J Obstet Gynaecol.

[44] Fitbit development: web API. Fitbit.

[45] Fitbit development: authorization. Fitbit.

[46] FHIR Specification v3.0.2. HL7 FHIR.

[47] McKnight PE, Najab J, Weiner IB, Craighead WE (2010). Mann-Whitney U Test. The Corsini Encyclopedia of Psychology, Volume 3.

[48] Student (1908). The probable error of a mean. Biometrika.

[49] Stein P, Falco L, Kuebler F, Annaheim S, Lemkaddem A, Delgado-Gonzalo R, Verjus C, Leeners B (2016). Digital womens health based on wearables and big data. Fertil Steril.

[50] Yu JL, Su YF, Zhang C, Jin L, Lin XH, Chen LT, Huang HF, Wu YT (2022). Tracking of menstrual cycles and prediction of the fertile window via measurements of basal body temperature and heart rate as well as machine-learning algorithms. Reprod Biol Endocrinol.

[51] Torous J, Lipschitz J, Ng M, Firth J (2020). Dropout rates in clinical trials of smartphone apps for depressive symptoms: a systematic review and meta-analysis. J Affect Disord.

[52] Pratap A, Neto EC, Snyder P, Stepnowsky C, Elhadad N, Grant D, Mohebbi MH, Mooney S, Suver C, Wilbanks J, Mangravite L, Heagerty PJ, Areán P, Omberg L (2020). Indicators of retention in remote digital health studies: a cross-study evaluation of 100,000 participants. NPJ Digit Med.

[53] Simblett S, Greer B, Matcham F, Curtis H, Polhemus A, Ferrão J, Gamble P, Wykes T (2018). Barriers to and facilitators of engagement with remote measurement technology for managing health: systematic review and content analysis of findings. J Med Internet Res.

[54] Ajayi KV, Wachira E, Onyeaka HK, Montour T, Olowolaju S, Garney W (2022). The use of digital health tools for health promotion among women with and without chronic diseases: insights from the 2017-2020 health information national trends survey. JMIR Mhealth Uhealth.

[55] Holko M, Litwin TR, Munoz F, Theisz KI, Salgin L, Jenks NP, Holmes BW, Watson-McGee P, Winford E, Sharma Y (2022). Wearable fitness tracker use in federally qualified health center patients: strategies to improve the health of all of us using digital health devices. NPJ Digit Med.

[56] Yu S, Alper HE, Nguyen AM, Brackbill RM, Turner L, Walker DJ, Maslow CB, Zweig KC (2017). The effectiveness of a monetary incentive offer on survey response rates and response completeness in a longitudinal study. BMC Med Res Methodol.

[57] Kawabe R, Chen CY, Morino S, Mukaiyama K, Shinohara Y, Kato M, Shimizu H, Shimoura K, Nagai-Tanima M, Aoyama T (2022). The relationship between high physical activity and premenstrual syndrome in Japanese female college students. BMC Sports Sci Med Rehabil.

[58] Samadi Z, Taghian F, Valiani M (2013). The effects of 8 weeks of regular aerobic exercise on the symptoms of premenstrual syndrome in non-athlete girls. Iran J Nurs Midwifery Res.

[59] Witkoś J, Hartman-Petrycka M (2021). The influence of running and dancing on the occurrence and progression of premenstrual disorders. Int J Environ Res Public Health.

[60] Cicek G (2018). The effect of regular aerobic exercises on premenstrual syndrome in sedentary women. Balt J Health Phys Act.

[61] El-Lithy A, El-Mazny A, Sabbour A, El-Deeb A (2015). Effect of aerobic exercise on premenstrual symptoms, haematological and hormonal parameters in young women. J Obstet Gynaecol.

[62] Maged AM, Abbassy AH, Sakr HRS, Elsawah H, Wagih H, Ogila AI, Kotb A (2018). Effect of swimming exercise on premenstrual syndrome. Arch Gynecol Obstet.

[63] Fuller D, Colwell E, Low J, Orychock K, Tobin MA, Simango B, Buote R, Van Heerden D, Luan H, Cullen K, Slade L, Taylor NGA (2020). Reliability and validity of commercially available wearable devices for measuring steps, energy expenditure, and heart rate: systematic review. JMIR Mhealth Uhealth.

