# Supplementary material for: Feasibility Study on Menstrual Cycles With Fitbit Device (FEMFIT): Prospective Observational Cohort Study
Source: JMIR Mhealth Uhealth. 2024 Mar 12;12:e50135. doi: 10.2196/50135 (PMC10966447; doi:10.2196/50135)
Supplement: Multimedia Appendix 2 [file mhealth_v12i1e50135_app2.docx]

**
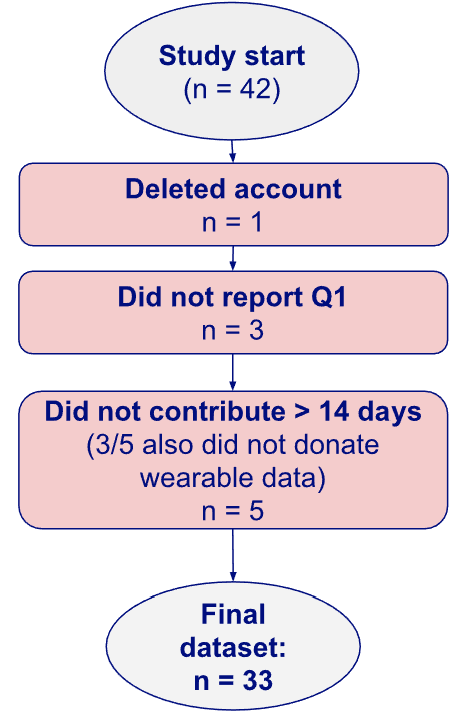
**

**Figure S1**: Flowchart visualizing participant drop-out during the study period. n = number.

| **Questionnaire** | **Q1** | **Q2** | **Q3** | **Q4** |
| --- | --- | --- | --- | --- |
| **Cadence** | **1x in week 1 of study** | **1x/week for 12 weeks** | **daily from day 13 to day 5** | **1x in week 12 of study** |
| **Content** | - Baseline characteristics - Relevant gynecological characteristics - 1st day of last menstrual cycle | Physical and mental well-being | - PMS symptoms - Questions about bleeding | App evaluation |

**Figure S2**: Cadence and content of digital questionnaires provided in study application.


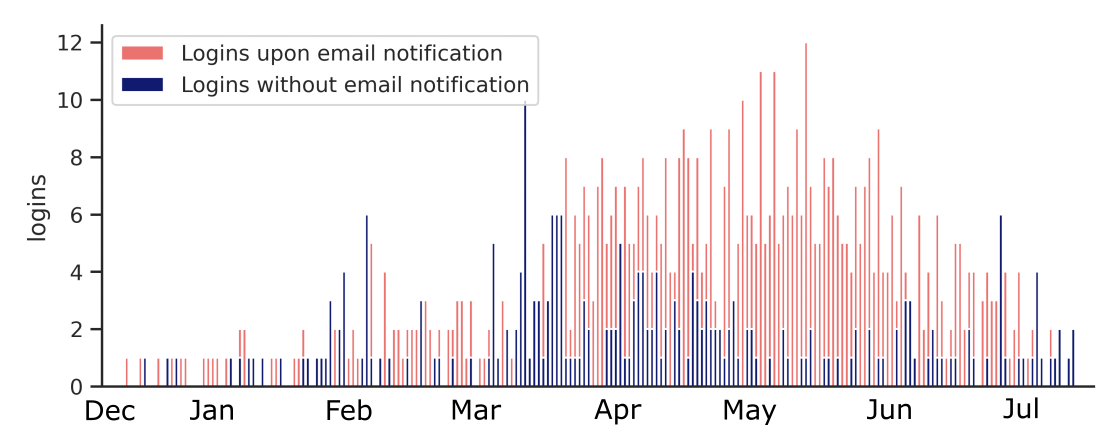


**Figure S3**: Barplot representing count of logins per day as measured using the Matomo Business analytics tool. Data was only collected for individuals that accepted tracking of cookies. Logins upon email notification contain all logins that directly followed the link to the study app provided in one of the frequent email notifications.


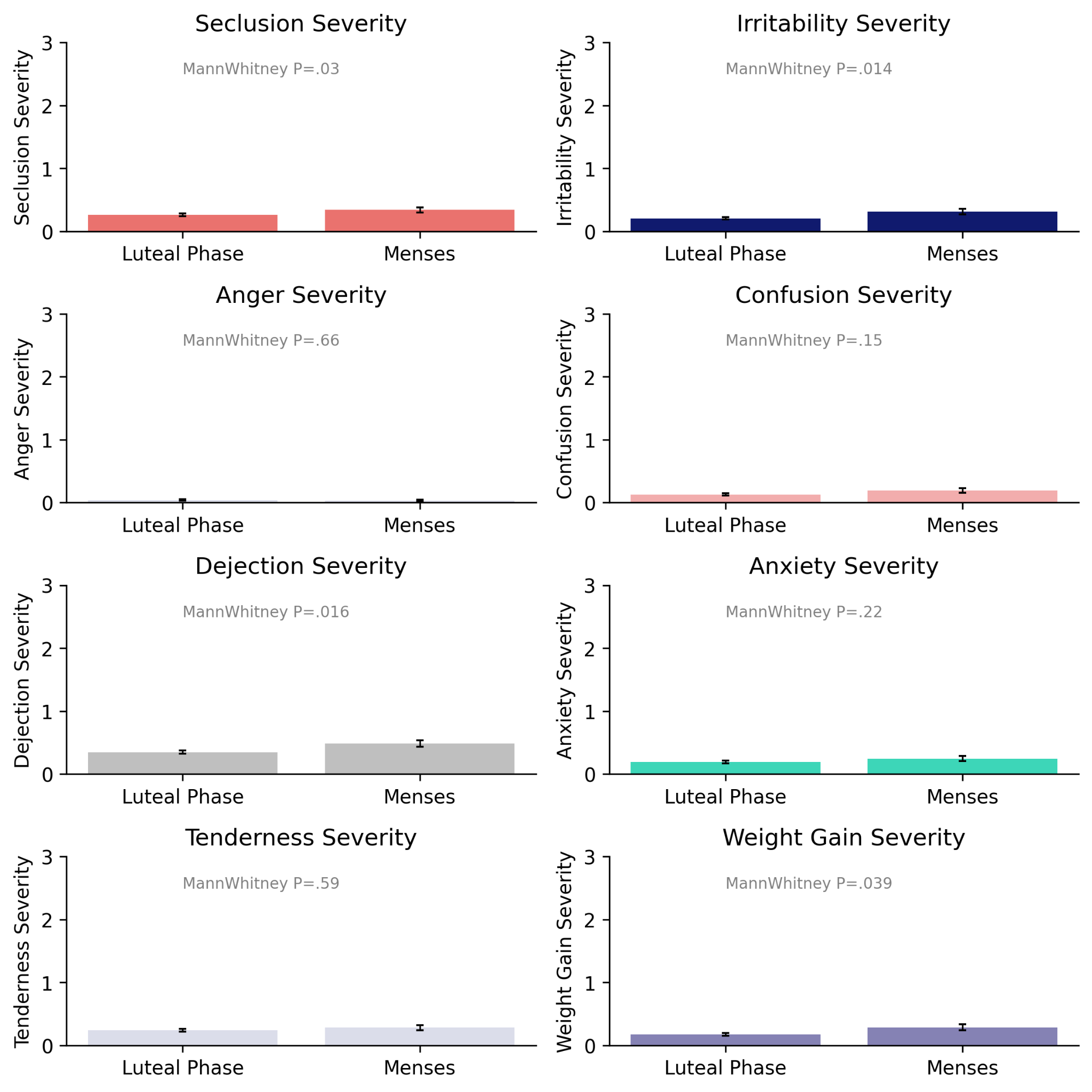


**Figure S4** Symptom severity in luteal phase compared to menses. Barplots show the mean rating for symptom severity on a scale from 0 to 3 from 0 = no symptoms to 3 = very strong symptom severity. Error bars reflect the standard error.


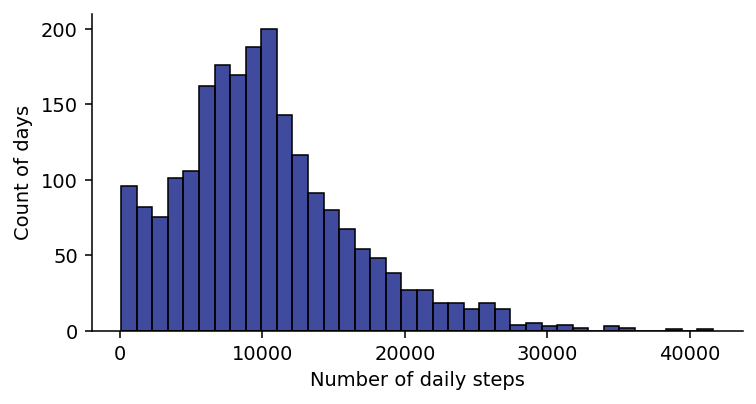


**Figure S5** Distribution of overall recorded total number of steps per day.


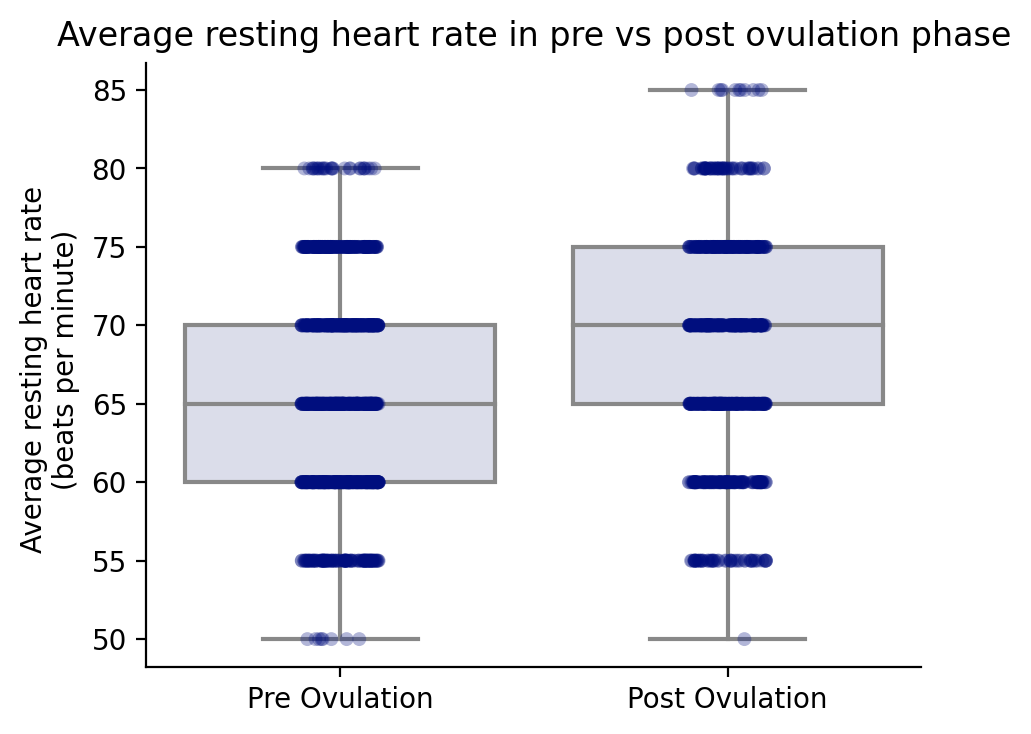


**Figure S6** Average resting heart rate in pre vs post ovulatory phase. Boxplots comparing the average resting heart rate in beats per minute on days before the ovulation (pre ovulation) with days after ovulation (post ovulation). Each dot represents one average resting heart rate measurement of one study participant.
